# Comparison of the Quality of Echocardiography Imaging Between the Left Lateral Decubitus and Supine Positions

**DOI:** 10.7759/cureus.31835

**Published:** 2022-11-23

**Authors:** Jakob Ottenhoff, Matthew Hewitt, Nardos Makonnen, Matthew Kongkatong, Christopher D Thom

**Affiliations:** 1 Department of Emergency Medicine, University Of Virginia Health System, Charlottesville, USA; 2 Department of Emergency Medicine, University of Virginia Health System, Charlottesville, USA

**Keywords:** cardiology, point of care ultrasound, emergency medicine, imaging quality, echocardiography

## Abstract

Introduction

It is commonly taught that positioning the patient in the left lateral decubitus (LLD) position will improve transthoracic echocardiography (TTE) image quality. Despite this, no previous studies have been performed that study this practice. Our goal was to quantify the difference in image quality of TTE views between the supine and LLD positions.

Methods

This was a prospective study in a single academic Emergency Department (ED) of a convenience sample of 30 patients. Three separate ED physicians performed TTE views in both the supine and LLD position on each patient. The order of position was randomized. Images were then reviewed on a previously validated TTE image quality scale by two blinded ED physicians with specialized training in ultrasound. The scale used a 0 to 5 (highest quality) metric for quality assessment. Interpretability of right ventricular and left ventricular function was also assessed.

Results

The mean image quality for the supine position was 2.85 (standard deviation {SD} 1.1) and 3.05 (SD 1.2) for the LLD position (p=0.044). In the subset of parasternal and apical windows, the mean quality for the supine position was 2.87 (SD 1.1) and 3.23 (SD 1.1) for the LLD position (p=0.003). The number of studies in which right ventricular function was interpretable was significantly higher in the LLD position (62% versus 42%, p=0.044).

Conclusions

There was a statistically significant increase in image quality when TTE was performed in the LLD position as compared to supine. This was especially pronounced in the apical four and parasternal windows.

## Introduction

Transthoracic echocardiography (TTE) is a commonly performed diagnostic test within several fields of medicine. It has seen rapid growth in the area of point-of-care ultrasound within emergency medicine, anesthesia, and critical care medicine. It has also become an integral component of the training in these specialties [1,2]. Point-of-care TTE affords the bedside physician the ability to garner time-sensitive and actionable information that would otherwise be inaccessible within a short time frame. This can provide critical diagnostic information and identify pathology that is immediately actionable, such as pericardial tamponade or massive pulmonary embolism [3,4]. It can also serve to guide resuscitative efforts on critically ill patients [5,6]. 

One of the difficulties inherent in TTE, whether performed by an echocardiography technician or a physician utilizing point-of-care ultrasound, is the acquisition of high-quality images. The heart can be difficult to image secondary to the chest wall tissue, the ribs, and the air present within the lungs. Obesity in particular can result in a significant reduction in image quality secondary to signal attenuation [7]. These factors can be a barrier to high quality imaging and can subsequently result in a reduction of useful diagnostic information from the TTE. Both global visual assessment and quantitative measurements such as the assessment of regional systolic function and chamber volume quantification can be greatly affected by poor image quality [8,9]. TTE image quality has also been identified as a factor associated with the reliability of echocardiographic measurements between expert readers [10]. Given the importance of image quality within echocardiography, the European Association of Cardiovascular Imaging recommends that the image quality of a TTE should be regularly reported on the TTE in a standardized fashion [11].

Patient positioning can be an important tool in improving TTE image quality. The American Society of Echocardiography (ASE) recommends placement in the left lateral decubitus (LLD) position for the apical and parasternal windows [12]. This position brings the heart into closer contact with the chest wall and thus closer to the ultrasound transducer [13]. In addition, the left arm can be placed above the head to increase the distance between the ribs and thus the intercostal space available for imaging [14]. The American College of Emergency Physicians (ACEP) also recommends the LLD position when possible, but notes that the clinical situation often limits the patient to stay in the supine position [15]. While these recommendations for body positioning are often taught and practiced, there has been no published quantitative evidence regarding the improvement of TTE views in the LLD position. Image quality differences between body positions in transesophageal echocardiography have been quantitatively investigated [16]. However, to our knowledge, there is no existing scientific evidence to support or refute a change in the quality of TTE imaging between the LLD and supine body position. We sought to quantitatively investigate the difference in image quality of standard TTE views between the supine and LLD positions using a previously validated echocardiography image scoring tool.

## Materials and methods

Study design and setting 

This was a convenience sample of patients in the Emergency Department (ED) setting at one large academic tertiary care center. The study protocol received approval through the University of Virginia Institutional Review Board, #210209, prior to the enrollment of any patients. The study period was from July 2021 to January 2022. Inclusion criteria included any ED patient 18 years of age or older who could provide written informed consent and who was able to change positions between supine and LLD. These were patients with a variety of illnesses who were waiting in the ED for their workup to conclude. Exclusion criteria included pregnant patients, hemodynamically unstable patients, and patients on spinal precautions. One emergency ultrasound fellow and two postgraduate year 3 emergency medicine resident physicians enrolled patients and performed the imaging. Each of these enrolling physicians had previously performed at least 25 peer-reviewed echocardiography studies and were considered competent in point-of-care echocardiography in accordance with ACEP training criteria [17]. Thirty patients were enrolled in the study.

Scanning protocol and interpretation

The enrolling physician used a random number generator to randomize patients to receive supine first or LLD first echocardiography. The enrolling physician then placed the patient in the first position and proceeded to obtain the standard echocardiographic views in an ordered fashion (e.g. parasternal long axis {PSLA}, parasternal short axis {PSSA}, apical four-chamber {A4C}, subcostal, and inferior vena cava {IVC}). While at the A4C window, the physician then took the measurements of left ventricular ejection fraction (LVEF) utilizing Simpson’s volumetric method and the tricuspid annular plane systolic excursion (TAPSE) utilizing M-mode. The LVEF and TAPSE measurements were utilized as a marker of left ventricular (LV) function and right ventricular (RV) function assessment. The images and measurements were then repeated in the second position. 

Following image acquisition, two emergency ultrasound fellowship-trained ED physicians with Focused Practice Designation in Advanced Emergency Medicine Ultrasonography reviewed the echocardiogram images. They were blinded to the order of patient positioning. Image quality was rated via a previously validated echocardiography scoring scale that utilizes a 0 to 5 scale, where 0 is ‘not obtained’, 1 is ‘image quality too poor to permit meaningful interpretation’, 3 is ‘suboptimal image quality, but basic image interpretation possible’, and 5 is ‘good image quality, meaningful image interpretation easy’ [18]. Each window is rated separately (e.g. PSLA, PSSA, A4C, subcostal, and IVC) on this scale. The aforementioned echocardiography scale also includes a binary 0 or 1 score for the assessment of LV function and RV function, with 0 indicating ‘image quality does not permit meaningful interpretation’ and 1 indicating ‘image quality permits meaningful interpretation’. A standardized reporting form was utilized for this scoring by the two blinded ultrasound-trained ED physicians.

Statistical methods

Data analysis was performed using VassarStats statistical computation software. Continuous data such as patient age are expressed as a median with interquartile range (IQR), while categorical data such as patient sex are expressed as a frequency count with percentage. The Mann-Whitney U test was used to determine the statistical significance of the echocardiography quality scale score between the two body positions. The Fisher exact test was then used to determine the statistical significance of LV and RV interpretability differences between positions. Interrater reliability between the two physician echocardiography graders was assessed via the Cronbach alpha (α). 

## Results

Thirty emergency department patients were enrolled. One emergency ultrasound fellow and three emergency medicine third year residents performed the studies. The median age of the patients enrolled was 57 (IQR 39-68). Of these 43% were female and 57% male. Median body mass index (BMI) was 25 (IQR 22-33). Twenty percent of patients had previously known cardiac disease (e.g. coronary artery disease, congestive heart failure, known valvular disease, or prior cardiac surgery), while 20% had previously known pulmonary disease (e.g. asthma, emphysema, or pulmonary sarcoidosis).

**Figure 1:**
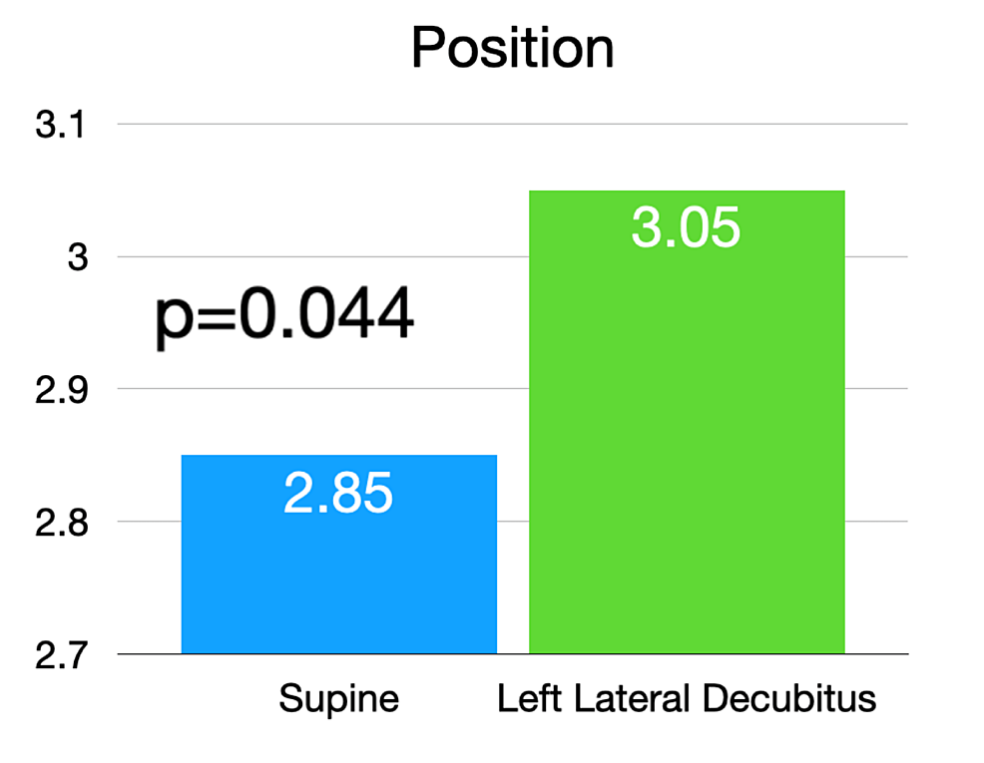
Average image quality score of all cardiac windows in the supine and left lateral decubitus positions

**Figure 2:**
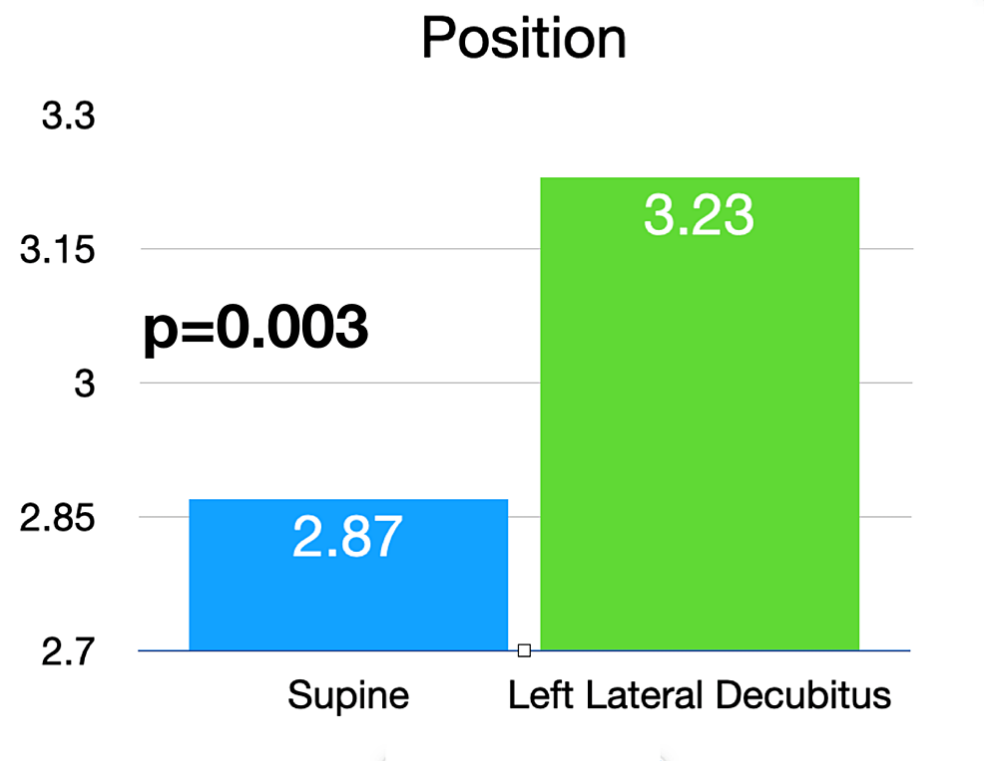
Average image quality score of apical and parasternal cardiac windows between the supine and left lateral decubitus windows

**Figure 3:**
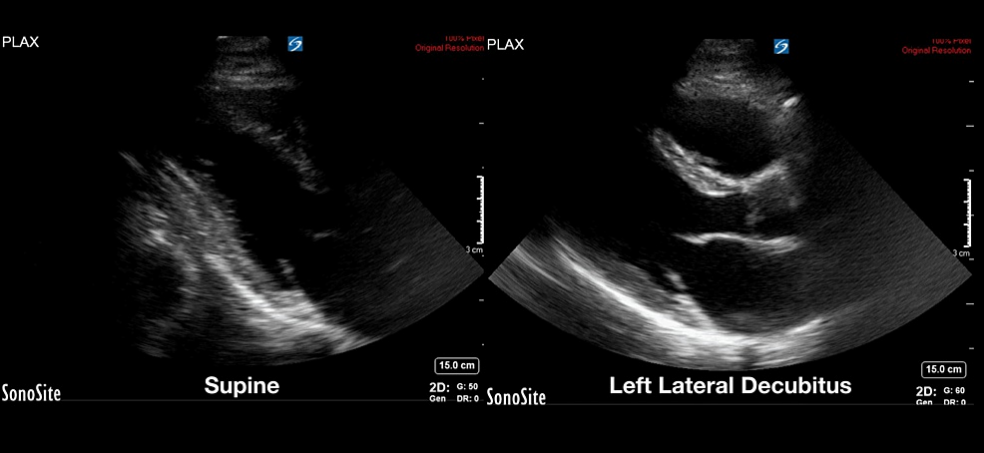
Example of image quality difference in the parasternal long window between the supine and left lateral decubitus positions within the same patient

**Figure 4:**
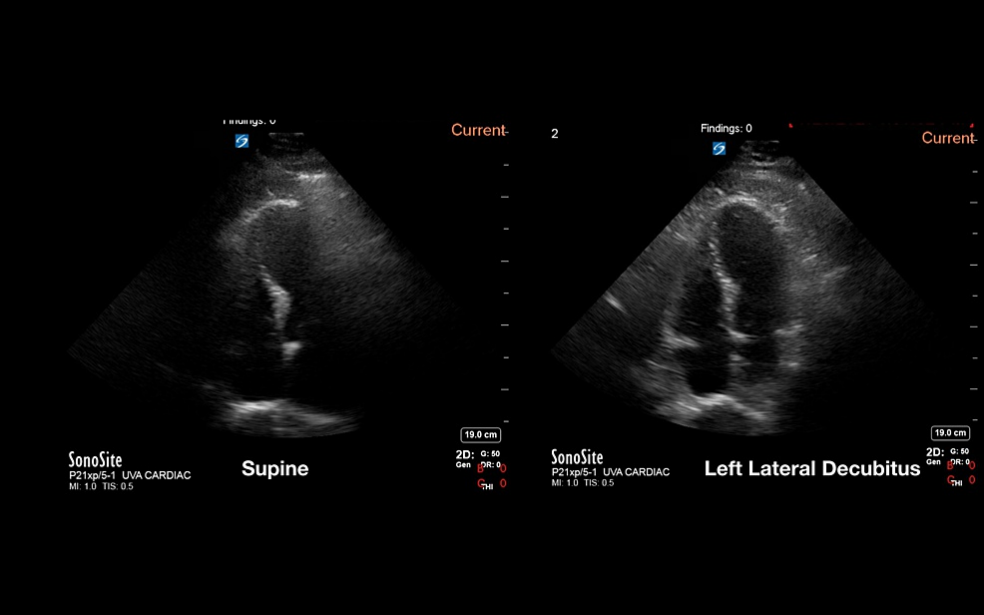
Example of image quality difference in the apical four window between the supine and left lateral decubitus positions within the same patient

Utilizing the previously validated echocardiography quality score, the pooled data from the two-blinded image reviewers demonstrated an average image quality for the supine position of 2.85 (SD 1.1) and an average image quality for the LLD position of 3.05 (SD 1.2). This was statistically significant with a p-value of 0.044 (Figure 1). Given that the LLD position is thought to be primarily advantageous in the parasternal and apical windows [11], the three views of the parasternal long, parasternal short, and apical four chamber were analyzed separately. In this subanalysis, the pooled data from the two reviewers showed an average supine position image score of 2.87 (SD 1.1) and and average LLD position score of 3.23 (SD 1.1), with a p-value of 0.003 (Figure 2). Examples of echocardiographic images taken from the same patient in the two different body positions are shown in Figures 3, 4. 

**Figure 5:**
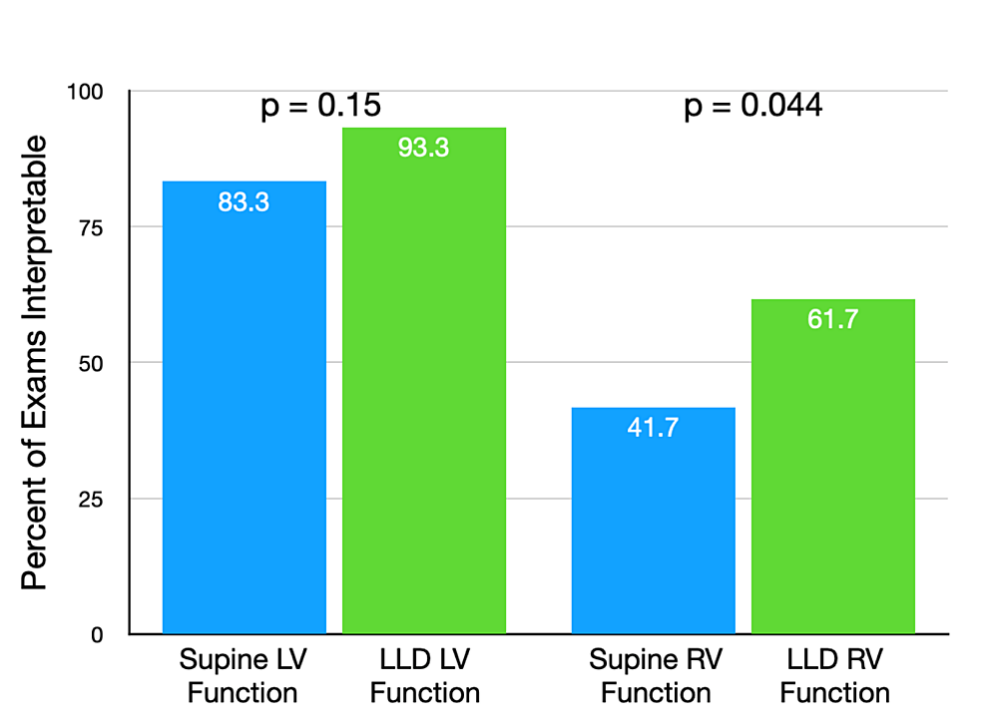
Percentage of exams with interpretable left ventricular and right ventricular function LV: left ventricular, LLD: left lateral decubitus, RV: right ventricular

**Figure 6:**
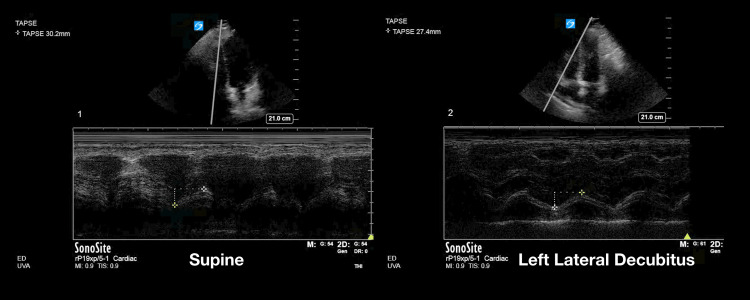
Example of image quality difference in the tricuspid annular plane systolic excursion (TAPSE) between the supine and left lateral decubitus positions within the same patient

**Figure 7:**
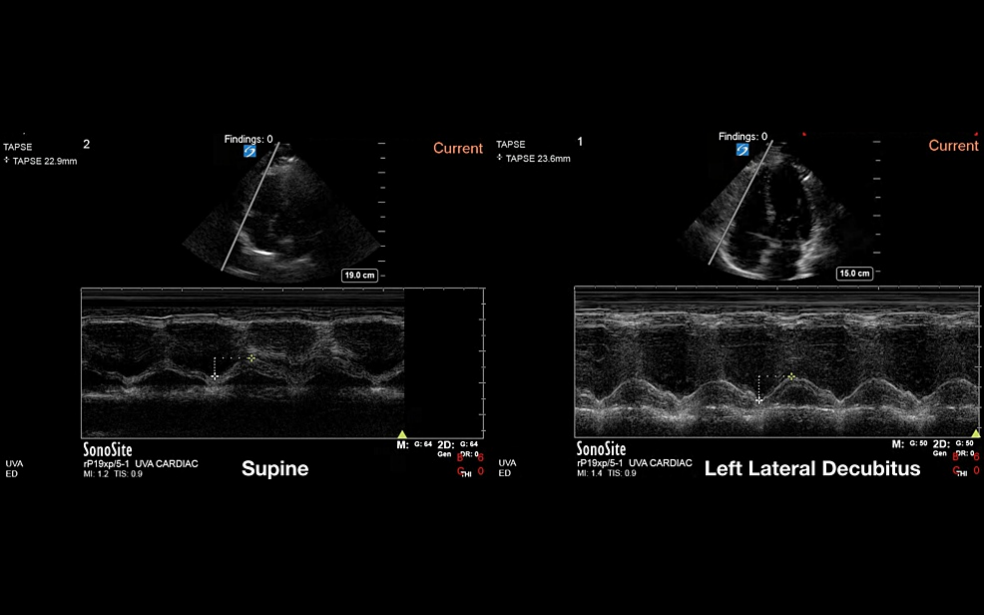
Example of image quality difference in the tricuspid annular plane systolic excursion (TAPSE) between the supine and left lateral decubitus positions within the same patient

In addition to the above, the quality rating score also included a section for whether the study was of high enough quality to allow for LV function and RV function to be assessed. The pooled data from the two reviewers showed that 83.3% of studies taken in the supine position were of high enough quality to allow for LV function interpretation, while this was 93.3% of studies in the LLD position (p = 0.15). RV function was interpretable in 41.7% of the supine studies and 61.7% of the LLD studies (p = 0.044). These results for LV and RV functions are shown in Figure 5. Echocardiographic images of TAPSE measurement in the two different positions are shown in Figures 6, 7. 

There were nine patients who had a BMI greater than 30 in the cohort. Within this subgroup, the average pooled image quality was 2.52 (SD 0.9) in the supine position and 2.84 (SD 0.9) in the LLD position (p = 0.134). 

Analyzing data between different echocardiography windows, we noted that the average image quality for the subcostal view in both positions was 2.67 (SD 1.11), while it was 3.13 (SD 1.12) in the parasternal long view (p = 0.003). This is congruent with a large study recently performed on echocardiography quality comparing subcostal and parasternal long views, wherein image quality was higher overall in parasternal long views [19]. The authors in this study suggest that perhaps the parasternal long view is better suited for usage during cardiopulmonary resuscitation given this. 

Interrater reliability among image reviewers was α=0.85 for the overall image quality assessment, indicating good agreement between the two reviewers.

## Discussion

To our knowledge, this is the first study that seeks to quantify the difference in image quality between standard transthoracic echocardiography views taken in the supine position and in the LLD position. It is longstanding teaching that the quality of echocardiographic images will improve in the LLD position for the PSLA and apical four chamber views secondary to the heart moving closer in proximity to the chest wall [20,21]. This recommendation is included by the ASE for the PSLA and apical four chamber positions [12]. However, there has been no prior quantification or verification of this premise in the literature. 

Previous work has been done that demonstrates certain TTE measurements can differ between supine and LLD body positions, including M-mode assessment in mitral valve prolapse and the measurement of the size of the right ventricular outflow tract [22,23]. Indeed, the change in body position can itself cause a physiologic change in cardiac function and valvular assessment [24]. It is thus prudent to keep the potential effects of positional changes in mind when acquiring and interpreting TTE images. In addition, it has been shown that image quality in TTE can affect measurement values and reproducibility of interpretations between individuals [8-10].

Our data showed that overall image quality did improve in the LLD position as compared with supine, particularly in the PSLA and apical four chamber views. In addition, there was a statistically significant increase in RV function interpretability when images were obtained in the LLD position. There was an increase in LV function interpretability as well, but this did not reach statistical significance. Our subgroup analysis of BMI greater than 30 patients demonstrated a higher image quality score in the left lateral decubitus position, though this did not reach statistical significance. Given our limited number of patients within this higher BMI cohort, more studies should be considered to explore the effect of the position change in this population. While not all patients can be positioned in the LLD position [15], our study does provide evidence that this practice may be beneficial to consider, particularly when faced with suboptimal or uninterpretable imaging quality.

Limitation

Our study had several important limitations. We utilized a convenience sample of ED patients who were available for ultrasound imaging and willing to participate in the research. These individuals did not have a specific indication for a transthoracic echocardiogram and thus the data generated here might not be immediately applicable to patients with a clinical indication for a transthoracic echocardiogram. Our study subjects also were variable in whether overall imaging quality was high or low. Additionally, there were only nine patients whose BMI was >30, which is a patient population where one might suspect a high utility for LLD positioning. Future studies could target specific patient subtypes such as this, as well as others that might be likely to have difficult TTE windows.

An additional limitation is the ED physicians performing the echocardiogram images were not blinded to the study design. This could have introduced bias in image capture. However, attempting to blind these individuals would likely have been unsuccessful given the nature of the study design and the need to capture each TTE window in two different body positions sequentially. Importantly, the two ultrasound fellowship-trained ED physicians who were interpreting the images were blinded to patient position and demonstrated good interrater reliability in their assessments.

The TTE image quality scale utilized was felt appropriate for this study design and had been previously validated in the literature. It should be noted that it was used as a competency assessment tool in the derivation study [18], which is somewhat different than our purpose here. However, it assessed competency through the evaluation of the quality of echocardiogram images on a reliable and clear scale that demonstrated good interrater reliability. For these reasons, this scale was felt appropriate and applicable to our study question. 

Finally, our study did not compare quantitative echocardiography measurements between the two positions with cardiology-performed echocardiography measurements. Further study could seek to explore which measurements are most likely to have increased accuracy when the ED physician repositions them to the LLD position. 

## Conclusions

Our pilot study is the first to demonstrate quantitative improvement in image quality when TTE images were obtained in the LLD position as compared to the supine position. This provides evidence for this commonly referenced teaching practice. In addition, we found an increased number of images in the LLD position that had image quality sufficiently high to allow for RV function interpretation. Future investigation to identify subsets of patients who benefit the most from imaging in the LLD position could be fruitful, as could investigations into which TTE measurements are most benefited from the increase in image quality. 
